# Molecular Mechanisms of Antipsychotic Drug-Induced Diabetes

**DOI:** 10.3389/fnins.2017.00643

**Published:** 2017-11-21

**Authors:** Jiezhong Chen, Xu-Feng Huang, Renfu Shao, Chen Chen, Chao Deng

**Affiliations:** ^1^School of Biomedical Sciences, The University of Queensland, St Lucia, QLD, Australia; ^2^School of Medicine, University of Wollongong, Wollongong, NSW, Australia; ^3^Illawarra Health and Medical Research Institute, Wollongong, NSW, Australia; ^4^Faculty of Science, Health, Education and Engineering, GeneCology Research Centre, University of the Sunshine Coast, Maroochydore, QLD, Australia

**Keywords:** antipsychotics, diabetes mellitus, metabolic disorders, insulin resistance, obesity, pancreatic beta cell, apoptosis

## Abstract

Antipsychotic drugs (APDs) are widely prescribed to control various mental disorders. As mental disorders are chronic diseases, these drugs are often used over a life-time. However, APDs can cause serious glucometabolic side-effects including type 2 diabetes and hyperglycaemic emergency, leading to medication non-compliance. At present, there is no effective approach to overcome these side-effects. Understanding the mechanisms for APD-induced diabetes should be helpful in prevention and treatment of these side-effects of APDs and thus improve the clinical outcomes of APDs. In this review, the potential mechanisms for APD-induced diabetes are summarized so that novel approaches can be considered to relieve APD-induced diabetes. APD-induced diabetes could be mediated by multiple mechanisms: (1) APDs can inhibit the insulin signaling pathway in the target cells such as muscle cells, hepatocytes and adipocytes to cause insulin resistance; (2) APD-induced obesity can result in high levels of free fatty acids (FFA) and inflammation, which can also cause insulin resistance. (3) APDs can cause direct damage to β-cells, leading to dysfunction and apoptosis of β-cells. A recent theory considers that both β-cell damage and insulin resistance are necessary factors for the development of diabetes. In high-fat diet-induced diabetes, the compensatory ability of β-cells is gradually damaged, while APDs cause direct β-cell damage, accounting for the severe form of APD-induced diabetes. Based on these mechanisms, effective prevention of APD-induced diabetes may need an integrated approach to combat various effects of APDs on multiple pathways.

## Introduction

Antipsychotic drugs (APDs) are widely prescribed to control schizophrenia and bipolar disorders, as well as other mental disorders including dementia, major depression, and even drug addiction (Fraguas et al., 2011; Leucht et al., 2013; Zhang J.-P. et al., 2013; Samara et al., 2016). Typical APDs (also called first generation APDs) such as chlorpromazine, perphenazine and haloperidol were introduced to clinics more than 60 years ago. Therapeutic effects of typical APDs are mediated largely through potent blockage of dopamine D2 receptors (Ginovart and Kapur, 2012), which also cause extra-pyramidal symptoms (EPS) side-effects (Stahl, 2003). Since the 1990s, a number of atypical APDs (also called 2nd generation APDs) including olanzapine, clozapine and risperidone have been approved by the FDA, and are now widely used as first line APDs due to their improved tolerability and reduced EPS compared with typical APDs (Leucht et al., 2009; Zhang J.-P. et al., 2013). In addition, clozapine has better outcomes in treatment-resistant schizophrenia (Lewis et al., 2006; Samara et al., 2016). Besides blockage of D2 receptors, atypical APDs target multiple neuroreceptors such as serotoninergic 5-HT2A/5-HT2C, histaminergic H1 and muscarinic M3 receptors (Correll, 2010). Although typical APDs have been reported causing a certain degree of metabolic disorders, atypical APDs, particularly clozapine and olanzapine, can cause much worse metabolic side-effects including body weight gain, obesity, hyperlipidaemia, insulin resistance, hyperglycaemia and diabetes (Foley and Morley, 2011; De Hert et al., 2012; Deng, 2013; Lipscombe et al., 2014; Stubbs et al., 2015). Since psychiatric patients often face chronic and even life-time APD treatment, these side-effects are major considerations in APD medication (De Hert et al., 2012; Deng, 2013). Schizophrenia patients with APD treatment have 2.5 times higher risk of developing type 2 diabetes according to a recent meta-analysis which examined 25 studies, including 145,718 individuals with schizophrenia (22.5–54.4 years) and 4,343,407 controls (Stubbs et al., 2015). It is noteworthy that over the last decade APD prescriptions in children and adolescents have sharply increased (Memarzia et al., 2014; Steinhausen, 2015). Recent studies have shown that APDs cause not only greater weight gain in children/adolescents than in adults but also significant risk of type 2 diabetes, which has been largely underestimated (Maayan and Correll, 2011; Samaras et al., 2014; Pramyothin and Khaodhiar, 2015; Sohn et al., 2015). These severe side-effects have a devastating impact on life quality, and is a key risk for severe health complications, including cardiovascular disease, stroke, and premature death (Foley and Morley, 2011; Lin et al., 2014; Wu et al., 2015). Understanding how diabetes develops in patients treated with APDs and preventing APD-induced diabetes will improve medication compliance.

## Antipsychotic-induced diabetes

Diabetes is characterized by hyperglycaemia (fasting plasma glucose ≥126 mg/dL [7 mmol/L]) due to insufficient insulin production or insulin resistance (Kahn et al., 2014). Insulin is well known to promote glucose metabolism to produce energy. Diabetes is commonly classified into two types; type 1 is due to auto-immune damage to pancreatic β-cells, leading to a complete deficiency of insulin; type 2 is caused by insulin resistance, i.e., the cells do not respond to insulin stimulation, as well as β-cell dysfunction (Kahn et al., 2014). There is a chronic development from insulin resistance to diabetes. In the case of insulin resistance, insulin secretion from β-cells can increase 4-fold while β-cell mass can increase 2-fold (Kahn et al., 2006). Therefore, blood glucose levels can still be maintained at normal levels, however, if β-cells fail to increase insulin secretion, diabetes will occur.

APDs have been shown to cause both diabetes and hyperglycaemic emergencies. Chronic administration of APDs is known to cause diabetes, which has been demonstrated in both epidemiological investigations in patients and in animal studies (Boyda et al., 2010a; Sohn et al., 2015; Stubbs et al., 2015). A recent study of 307 patients with psychotic illnesses showed that olanzapine caused type 2 diabetes (17%), obesity (48%), dyslipidaemia (35%) and hypertension (32%) with mean treatment duration of 7.6 years, while other APDs also induced similar side-effects (Reed et al., 2014). Another study has shown that APDs caused 37% prediabetes and 10% diabetes (Manu et al., 2012). Furthermore, the European First-Episode Schizophrenia Trial (EUFEST) reported a 20–30% incidence rate of hyperglycemia after 1 year of treatment with olanzapine, quetiapine and ziprasidone, but no significant differences between these APDs (Fleischhacker et al., 2013). Comparison of 28,858 APD users with 14,429 controls showed that the risk of diabetes increased 3-fold in children and adolescents treated with APDs (Bobo et al., 2013). This APD-induced diabetes has been confirmed in animal models; olanzapine and clozapine have been shown to decrease the plasma level of insulin and to cause hyperglycaemia and insulin resistance in rats (Chintoh et al., 2009; Boyda et al., 2010a; Liu et al., 2017).

The major complication of diabetes is heart disease. APDs significantly increased acute myocardial infarction with adjusted odds ratio of the risk 2.52 (95% CI = 2.37–2.68) (Lin et al., 2014). Citrome et al. examined diabetes and cardiovascular disease incidence in a large number of patients who used APDs including aripiprazole, olanzapine, risperidone, quetiapine, and ziprasidone (Citrome et al., 2013). It was found that these drugs caused diabetes at similar rates but olanzapine, risperidone and quetiapine caused more cases of cardiovascular disease than aripiprazole and ziprasidone (Citrome et al., 2013).

Recent attention has also been focused on APD-induced hyperglycaemic emergencies. Patients treated with atypical APDs have ~10 times higher risk in developing diabetic ketoacidosis (Polcwiartek et al., 2016). Lipscombe and colleagues conducted a multicenter retrospective cohort study including 725,489 patients to investigate hyperglycaemic emergencies (hyperglycaemia, diabetic ketoacidosis, hyperosmolar hyperglycaemic state) experienced by new users of risperidone, olanzapine, and other typical and atypical APDs (Lipscombe et al., 2014). The results showed that hyperglycaemic emergencies were 1 per 1,000 persons in patients aged 18–65 and 2 per 1,000 persons in those older than 65. The events were much more frequent in patients with pre-existing diabetes (6–12 per 1,000 persons) (Lipscombe et al., 2014). It was similar in the risk of hyperglycaemic emergencies with initiation of olanzapine vs. risperidone.

In brief, both clinical and animal studies have shown that APDs can cause serious glucometabolic side-effects including hyperglycaemic emergency, insulin resistance, hyperglycaemia and type 2 diabetes, which is a major risk for cardiovascular disease and premature death. It is worth to note that the risk of type 2 diabetes in children and youths treated with APDs has been underestimated (Samaras et al., 2014). Understanding the underlying mechanisms will be important for preventing and treating these side-effects and thus improving the clinical outcomes of APDs.

## Mechanisms for antipsychotic-induced diabetes

Over the past 10 years, a number of studies have aimed to explore the potential mechanisms underlying APD-induced diabetes. Based on recent progress, we summarized the findings into three molecular mechanisms for explaining APD-induced diabetes: (1) insulin resistance due to the direct effect of APDs, (2) APD-caused insulin resistance through obesity, and (3) APD-induced β-cell dysfunction and apoptosis.

### Mechanism 1: insulin resistance due to direct effect of antipsychotics

Insulin, secreted by pancreatic β-cells, is the key hormone in promotion of glucose metabolism (Kahn et al., 2014). It increases the uptake of glucose by cells and thus maintains the homeostasis of blood glucose levels. Insulin resistance refers to the situation where the target cells lose response to insulin stimulation and thus reduce glucose uptake (Kahn et al., 2014). Increased blood glucose level are mainly caused by insulin resistance in the skeletal muscles, and also in the hepatic, renal and adipose tissue (Kahn et al., 2014). Of these, the main site for glucose utilization is muscle tissue, and represents ~80% of glucose consumption (Fujii et al., 2005).

APD-induced insulin resistance could be independent of weight gain and increased food intake. It has been reported that, in patients within 3 months after initiation or switch to atypical APDs, new-onset gluco-metabolic abnormalities and diabetes was not associated with weight change and BMI (van Winkel et al., 2008). A recent study has shown that a single administration of olanzapine caused glucose metabolism change independent of obesity in healthy human subjects (Hahn et al., 2013). Aripiprazole has been shown to induce insulin resistance with metabolic changes where there is no weight gain or increase in food intake (Teff et al., 2013). Boyda et al. examined the acute effects (60, 180, or 360 min) of olanzapine, clozapine, risperidone and haloperidol on insulin resistance in rats at both low and high concentrations of clozapine (2 mg/kg; 20 mg/kg), olanzapine (1.5 mg/kg vs. 15 mg/kg), risperidone (0.5 mg/kg vs. 2.5 mg/kg) and haloperidol (0.1 mg/kg vs. 1.0 mg/kg) (Boyda et al., 2010b). Insulin resistance was evaluated through HOMA-IR (homeostasis model assessment index for insulin resistance) values in fasted rats and glucose clearance during a glucose tolerance test. Both olanzapine and clozapine produced significant dose and time dependent effects on fasting plasma glucose and insulin concentrations, HOMA-IR values, insulin resistance and glucose intolerance (Boyda et al., 2010b; Liu et al., 2017). However, risperidone and haloperidol also caused significant increases in fasting glucose and/or insulin levels at the high dose, 60 min post-drug administration (Boyda et al., 2010b).

**Figure 1:**
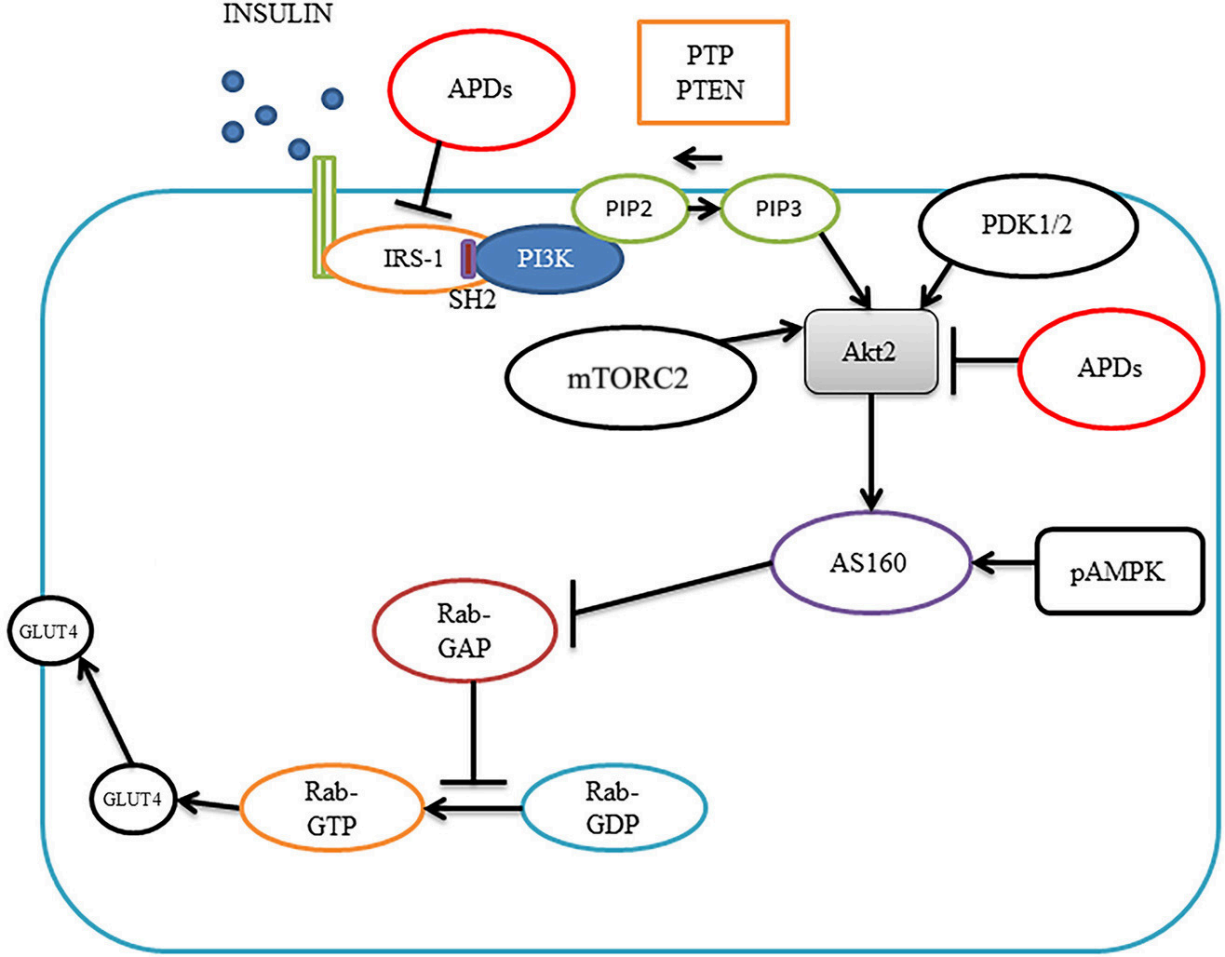
Insulin signaling pathways and antipsychotic effects. Insulin binds to insulin receptors to activate IRS1, leading to activation of PI3K, which converts PIP2 into PIP3, PIP3 brings Akt2 on to the membrane where PDK1/2 and mTORC2 phosphorylate Akt2. Akt2 activates AS160, which blocks Rab-GAP, leading to increased Rab-GTP, causing translocation of GLUT4 to the membrane for glucose transportation. Antipsychotics can diminish insulin-induced IRS-1 phosphorylation and inhibit Akt activity causing insulin resistance. Akt2, protein kinase 2; pAMPK, phosphor-AMP-activated protein kinase; APDs, antipsychotic drugs; GLUT4; glucose transporter type 4; IRS1, insulin receptor substrate 1; PI3K, phosphoinositide 3-kinase; PIP2, phosphatidylinositol (3,4)-bisphosphate; PIP3, phosphatidylinositol (3,4,5)-trisphosphate; PDK1/2, phosphoinositide-dependent kinase-1; mTORC2 mammalian target of rapamycin complex 2 and SH2, Src homology 2.

Insulin plays an important role in glucose consumable cells via the phosphoinositide 3-kinase (PI3K)/protein kinase B (PKB, also known as Akt) pathway (Figure 1). Insulin can bind to insulin receptors, leading to receptor autophosphorylation which in turn activates receptor tyrosine kinases that phosphorylate insulin receptor substrates (IRSs) including IRS1, IRS2, IRS3, IRS4, Gab1 (a member of the IRS1-like multisubstrate docking protein family) and Shc protein (Boucher et al., 2014). Phosphorylated IRSs activate PI3K by binding to its regulatory subunit via Src homology 2 (SH2) domains, increasing the conversion of phosphatidylinositol (3,4)-bisphosphate (PIP2) into phosphatidylinositol (3,4,5)-trisphosphate (PIP3). PIP3 can increase the activities of 3-phosphoinositide-dependent protein kinases 1/2 (PDK1/2). PIP3 also binds to Akt and this leads to translocation of Akt to the membrane, where Akt is phosphorylated by PDK1/2 and mammalian target of rapamycin complex 2 (mTORC2) and activated. Activated Akt increases glucose transporter type 4 (GLUT4) translocation to the membrane to increase glucose uptake via Akt substrate AS160 (Boucher et al., 2014). There are three Akt isoforms; 1, 2, and 3. The isoform regulating GLUT4 is Akt 2. GLUT4 is mainly located in adipose tissue and striated muscle (skeletal and cardiac) as well as the neurons of the hippocampus. Inhibition of Akt by siRNA has been shown to abolish the effect of insulin on glucose transport in 3T3-L1 adipocytes (Jiang et al., 2003). Insulin action is impaired in the liver, adipose tissue and muscle of Akt2 knockout mice (Cho et al., 2001). There are also two natural negative regulators in the pathway: protein tyrosine phosphatases (PTPs) and phosphatise and tension homolog deleted on chromosome 10 (PTEN); PTPs dephosphorylate insulin receptors and PTEN converts PIP3 into PIP2 (Boucher et al., 2014). Deficiencies in PTPs and PTEN have been shown to increase insulin sensitivity in mice (Boucher et al., 2014).

In type 2 diabetes patients, it has been shown that insulin-induced IRS-1 tyrosine phosphorylation, PI3K and Akt activities are decreased in skeletal muscles (Fröjdö et al., 2009). In adipose tissue, Akt2 phosphorylation is impaired in obese type 2 diabetes (Rondinone et al., 1999). In animal models, high fat-diet rats have defective insulin-induced GLUT-4 translocation in skeletal muscle which has been associated with decreased Akt activity (Tremblay et al., 2001). Therefore, abnormality of the insulin pathway in insulin target cells could be critical for the pathogenesis of diabetes.

APDs have been shown to inhibit Akt activity and thus cause insulin resistance in muscle cells (Engl et al., 2005). In L6 myotubes, olanzapine was shown to decrease glycogen content in a dose- and time- dependent fashion. Olanzapine diminished insulin-induced IRS-1 phosphorylation and abolished insulin-induced pPI3K, pAkt, and pGSK-3, while amisulpride, which does not cause diabetes, did not result in the above changes, indicating the importance of the insulin pathway in APD-induced diabetes (Engl et al., 2005). Olanzapine has been found to attenuate phosphorylation of insulin-like growth factor receptor (IGF-R) and IRS1 stimulated by insulin in wild-type fibroblast cells (Alghamdi et al., 2014). The mechanism has been considered to be olanzapine-induced membrane associated mammalian neuraminidase-3 (Neu3) and Neu 1 sialidase activity reduction. Alghamdi reported that Neu1 and matrix metalloproteinase-9 (MMP-9) could cross-talk in alliance with the neuromedin B G-protein coupled receptor (GPCR) to promote the insulin signaling pathway (Alghamdi et al., 2014). Clozapine has been reported to reduce insulin-stimulated glucose uptake in PC12 and in L6 cells, which was companioned with 40% decreased insulin effect on insulin receptor, 60% decrease in IRS1 tyrosine phosphorylation, and 40% decrease in insulin-stimulated Akt phosphorylation (Panariello et al., 2012). The inhibitory effect of APDs on Akt activity has also been reported in T-cells and glioblastoma cells (Shin et al., 2006; Chen M.-L. et al., 2011). However, the effects of APDs on the other elements of the insulin pathway such as PTPs and PTEN have not been studied and warrant further investigation.

### Mechanism 2: antipsychotic-caused insulin resistance through obesity

APDs are a well-known cause of obesity which is closely associated with diabetes. It has been repeatedly reported that APD-induced insulin resistance and diabetes are associated the increased weight gain, BMI and intra-abdominal adiposity, particularly in patients with chronic APD treatment (Bou Khalil, 2012; Manu et al., 2012). As many as 20–50% of APD treated patients are obese and have diabetes (Deng, 2013). Animal experiments also demonstrated that APDs can cause obesity, although there are some limitations (Boyda et al., 2010a; Weston-Green et al., 2011; van der Zwaal et al., 2014). One limitation is a failure to induce weight gain by clozapine in rodents in the majority of studies, although clozapine has been well established to cause dyslipidaemia, hyperglycaemia and insulin resistance in rodents (Boyda et al., 2010a; Liu et al., 2017), which provides a model to investigate the direct effects of clozapine on these metabolic parameters in rats without weight gain (Cooper et al., 2008; Liu et al., 2017). However, a recent study has reported that chronic clozapine treatment induced weight gain and fasting glucose levels in male rats (von Wilmsdorff et al., 2013). The most successfully established rodent model for APD-induced weight gain/obesity is by olanzapine (Weston-Green et al., 2011; van der Zwaal et al., 2014; Lord et al., 2017). Other APDs such as risperidone, sulpiride, haloperidol, and chlorpromazine have also been established to cause some degree of weight gain in rats and mice (Boyda et al., 2010a; Lian et al., 2015). There is also a sexual dimorphism in the obesogenic response to APDs, which is particularly clear in rodent models with male rats repeatedly reported less sensitive to APD-induced weight gain than females (Minet-Ringuet et al., 2006; Weston-Green et al., 2010; von Wilmsdorff et al., 2013). Although sex differences are less established in humans, females have been often observed with higher risks for weight gain and other metabolic side-effects (including increased insulin-resistance and higher plasma triglycerides) caused by APDs (Wu et al., 2007; Seeman, 2009; Weston-Green et al., 2010). Based on data from both clinical and animal studies, there are three stages in APD-caused obesity: during the first few months treatment in schizophrenia patients, APDs cause rapid weight gain (stage 1) followed by a steady weight gain for about 1 year (stage 2) to reach a plateau and then a stage (stage 3) for maintaining heavy weight (Zipursky et al., 2005; Pai et al., 2012; Deng, 2013). Similar developmental stages of APD-induced obesity have also been observed in rat models (Huang et al., 2006; He et al., 2014). Therefore, APD-caused obesity is a chronic process.

**Figure 2:**
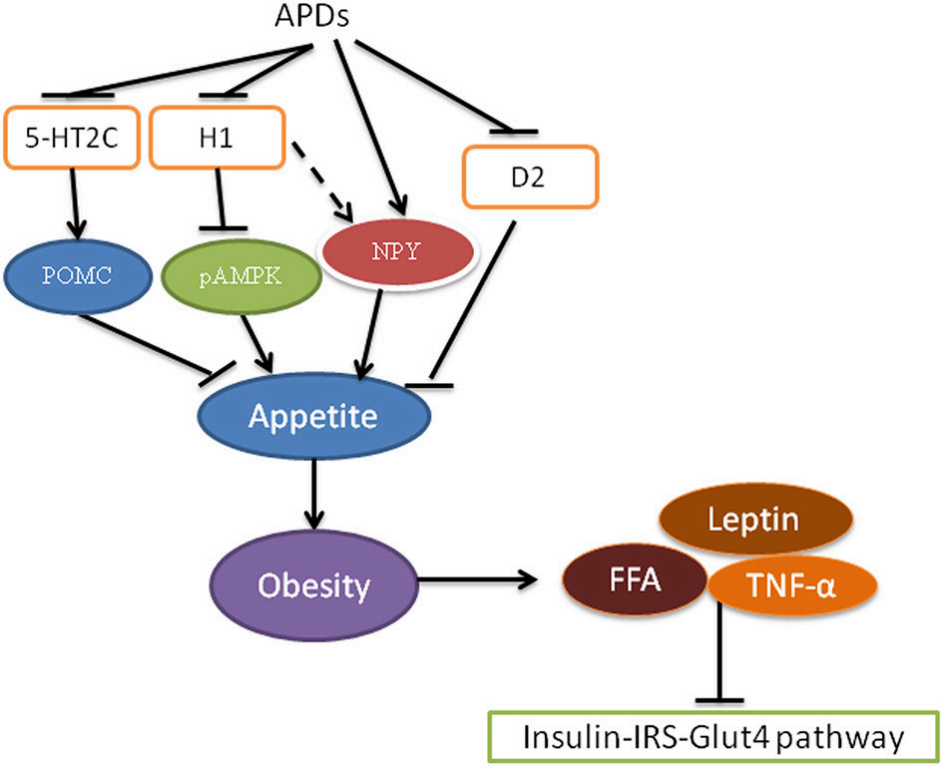
Antipsychotics cause insulin resistance via obesity. Antipsychotics block receptors 5-HT2C, histamine H1 and D2 receptors resulting in a decrease in POMC and an increase in NPY production, leading to increased appetite. Increased food intake results in obesity, which is associated with insulin resistance via increased FFA, leptin and TNF-α. pAMPK, phosphor-AMP-activated protein kinase; APDs, antipsychotic drugs; FFA, free fatty acids; D2, dopamine D2 receptor; 5-HT2C, Serotonin 5-HT2C receptor; NPY, neuropeptide Y; POMC, proopiomelanocortin; TNF-α, tumor necrosis factor alpha.

The mechanism for APD-caused obesity is considered to be increased appetite and food intake rather than decreased energy expenditure in the early stage of obesity development (Deng, 2013). APDs can act as an antagonist on serotonin 5-HT2C, histamine H1, and dopamine D2 receptors to increase appetite and thus increase food intake, leading to obesity (Figure 2; Matsui-Sakata et al., 2005; Han et al., 2008; Nasrallah, 2008; Kirk et al., 2009; Deng et al., 2010; Lian et al., 2016). It has been found that inhibition of 5-HT2C by olanzapine contributes to olanzapine-induced weight gain and hyperphagia in rodents (Kirk et al., 2009; Lord et al., 2017), while these effects were blunted in mice lacking 5-HT2C (*htr2c*-null mice) (Lord et al., 2017). 5-HT2C can promote anorexigenic proopiomelanocortin (POMC) neurons to decrease appetite (Lam et al., 2008, 2010; Lian et al., 2016). Indeed, olanzapine has been shown to decrease POMC expression in rats (Ferno et al., 2011; Weston-Green et al., 2012a; Lian et al., 2014a; Zhang et al., 2014a). Matsui-Sakata showed that H1 receptor blockage is the main reason for APD-induced obesity (Matsui-Sakata et al., 2005). APDs block H1 receptors and thus activate hypothalamic 5' AMP-activated protein kinase (AMPK), which stimulates appetite in rats (Kim et al., 2007; Han et al., 2008; Deng et al., 2010; He et al., 2013). It is interesting that an histamine H1 agonist (2-(3-trifluoromethylphenyl) histamine) is able to reverse increased hypothalamic AMPK activation and hyperphagia induced by olanzapine in rats (He et al., 2014). Fernø and colleagues have reported a decreased hypothalamic AMPK phosphorylation by subchronic olanzapine treatment and no effects after acute treatment of olanzapine or clozapine in rats (Ferno et al., 2011), however they measured AMPK phosphorylation 20 h after the final drug treatment that might explain their failures to detect the increased AMPK activation by APDs in this study. In fact, a further study by the same research group has proven the important role of hypothalamic AMPK activation in olanzapine-induced weight gain by means of adenovirus-mediated inhibition of AMPK in the arcuate nuclei of female rats with olanzapine depot exposure (Skrede et al., 2014). Furthermore, direct intracerebroventricular infusion of olanzapinehas been reported to induce hypothalamic AMPK activation and hepatic insulin resistance in rats (Martins et al., 2010). APDs have also been found to increase neuropeptide Y (NPY) expression (Lian et al., 2014a; Zhang et al., 2014a), which could be partially through blocking H1 receptors and reversed by H1 agonist (Lian et al., 2014a,b, 2016). The D2 receptor is associated with the reward system and thus appetite (Deng, 2013). APDs can bind and block D2 receptors to increase appetite (Nasrallah, 2008; Deng, 2013). Recently a study has shown that the gut microbiome plays an important role in APD-induced obesity in rats (Davey et al., 2013). In view of the importance of this microbiome in diet-induced obesity (Bäckhed et al., 2004, 2007; Turnbaugh et al., 2009), Davey et al. tested the role of this microbiome in olanzapine-induced obesity and found that use of antibiotics resulted in decreased fat accumulation in rats treated with olanzapine (Davey et al., 2013). Regarding to energy expenditure, similar to the sedative effects of APDs on humans, it has been widely reported that APDs such as olanzapine and risperidone decreased locomotor activities in both rats and mice (Fell et al., 2007; Cope et al., 2009; Weston-Green et al., 2011; Zhang et al., 2014b; Lian et al., 2015; Lord et al., 2017). Although a recent study found an increased heat production in mice treated with olanzapine (Lord et al., 2017), a previous study reported a decrease of metabolic rate in mice with olanzapine infusion (8 mg/kg/day) but not in mice with a lower dose (4 mg/kg/day) (Coccurello et al., 2009). Furthermore, a number of studies in rats reported that olanzapine reduced core body temperature or thermogenesis of brown adipose tissue (BAT) accompanied with decreased expression of functional thermogenic proteins, uncoupling protein1 (UCP1) and peroxisome proliferator-activated receptor gamma coactivator-1α (PGC-1α) of BAT (Stefanidis et al., 2009; van der Zwaal et al., 2012; Zhang et al., 2014b). Therefore, further studies are necessary to clarify the effects of APDs on thermogenesis.

Obesity is closely related to diabetes through insulin resistance and inflammation (Kahn et al., 2014; Verma and Hussain, 2017). In obesity, blood insulin levels are increased. However, in obese rats, the efficacy of insulin to stimulate Akt2 activity decreases in the muscle and liver, suggesting insulin resistance in these tissue (Kim et al., 2000). In obesity, increased blood levels of free fatty acids (FFA), tumor necrosis factor alpha (TNF-alpha) and leptin have been demonstrated to contribute to the development of type 2 diabetes (Verma and Hussain, 2017). Blood levels of FFA were increased in obesity due to an increased release from an increased size and number of adipocytes (Verma and Hussain, 2017). Increased FFA levels in turn inhibit insulin/IRS phosphorylation, leading to insulin resistance in insulin target cells such as skeletal muscle, the liver and endothelial cells (Kahn et al., 2006; Wilding, 2007). Decreased IRS phosphorylation causes decreased PI3K/Akt pathway activity and GLUT4 translocation (Wilding, 2007). Lowering the levels of FFA has been shown to increase insulin sensitivity in obese and non-obese diabetic subjects, indicating the important role of FFA in insulin resistance (Goodpaster and Coen, 2014). Exercise has been shown to reduce FFA and improve insulin sensitivity (Kwak, 2013; Malin et al., 2016).

Olanzapine has been shown to increase lipogenesis and thus increase FFA (Albaugh et al., 2011a). Recent studies showed that blood levels of FFA were significantly increased in rats after chronic administration of olanzapine (Davey et al., 2013; Liu et al., 2015, 2017). Jassim et al showed that a single injection of clozapine and olanzapine in rats increased serum FFA after 12–24 h (Jassim et al., 2012). A recent study in male rats showed that olanzapine could stimulate lipogenesis independent of weight gain, but with dosage-dependent effects (Ferno et al., 2015). All these studies support the view that APD-induced obesity could contribute to insulin resistance partially via increased blood levels of FFA.

TNF-alpha, which is increased in obesity, may also play a key role in the link between obesity and diabetes (Verma and Hussain, 2017). Both cell culture and animal studies have shown TNF-alpha to interfere with the insulin pathway causing insulin resistance (Nieto-Vazquez et al., 2008). Knockout of TNF-alpha decreased obesity-caused insulin resistance (Uysal et al., 1997). In addition, Interleukin 1 (IL-1), IL-6 and leptin are also increased in obesity and have been associated with insulin resistance (Verma and Hussain, 2017). Chronic treatment (46 days) with olanzapine has been reported to increase TNF alpha expression in adipose tissue and plasma and IL-1 expression in rat plasma (Victoriano et al., 2010). In addition, administration of olanzapine and other atypical APDs has been shown to increase blood levels of leptin and ghrelin, suggesting that leptin and ghrelin could also be a link between APD-induced obesity and diabetes (Jin et al., 2008; Albaugh et al., 2011b; Zhang Q. et al., 2013). As an adipocyte-derived hormone, adiponectin controls lipid and carbohydrate metabolism, in which a low adiponectin level is associated with obesity, insulin resistance and type 2 diabetes (Jin et al., 2008). Schizophrenia patients with metabolic disorders have been reported with a low adiponectin levels (Hanssens et al., 2008; Oriot et al., 2008; Chen P.-Y. et al., 2011; Sugai et al., 2012). Accumulated evidence has shown that treatment with atypical APDs clozapine and olanzapine decreases plasma adiponectin levels associated with weight gain, high fasting glucose, hypertriglyceridemia, insulin resistance induced by these drugs (Hanssens et al., 2008; Bai et al., 2009; Wampers et al., 2012; Klemettila et al., 2014), although it should be noted that some atypical APDs (e.g., risperidone) did not affect adiponectin levels (Murashita et al., 2007; Wampers et al., 2012).

### Mechanism 3: antipsychotic-induced β-cell dysfunction and apoptosis

Recent studies have paid attention to the role of β-cells in APD-induced diabetes. Pancreatic β-cells are the only type of cells that secrete insulin. The pathogenesis of diabetes involves the altered mass and function of β-cells (Kahn et al., 2014). It has been recognized that β-cell damage is necessary for the pathogenesis of type 2 diabetes (Kahn et al., 2014). Insulin resistance alone is regarded as insufficient to cause diabetes. According to β-cell damage, the development of diabetes is classified into five stages (Weir and Bonner-Weir, 2004). Stage 1 is compensation in which insulin secretion is increased and blood glucose levels are maintained at normal levels. Stage 2 is characterized by the mild rise of blood glucose levels to 5.0–6.5 mmol/L in which there are loss of β-cell mass and function. Stage 3 is early decompensation, in which maintenance of a normal range of blood glucose levels does not occur due to the inability of damaged β-cells to secrete insulin. Stage 4 is characterized by stable decompensation and significant β-cell damage. Stage 5 is major decompensation where profound reduction of β-cell mass and ketosis occurs. Therefore, protection of β-cells and promotion of human β-cell proliferation have been investigated in preclinical models to improve diabetes (Xiao et al., 2014; Shirakawa and Kulkarni, 2016).

APDs can cause β-cell damage directly and indirectly. The first evidence for APD-induced β-cell damage was from the studies of olanzapine-induced insulin resistance in canine models in comparison with a high-fat diet (Ader et al., 2005; Bergman and Ader, 2005). It was found that there was no expected β-cell compensation for olanzapine-induced insulin resistance (Ader et al., 2005; Bergman and Ader, 2005). This is different from insulin resistance induced by a high-fat diet which confers β-cell compensation, suggesting that olanzapine can cause β-cell damage. Chintoh et al also demonstrated that APDs caused β-cell damage in a rat model (Chintoh et al., 2009). It was found that acute treatment with clozapine and olanzapine at the dosages of 10 and 3 mg/kg respectively produced insulin resistance evidenced by decreased glucose infusion, increased hepatic glucose production and decreased peripheral glucose utilization (Chintoh et al., 2009). Clozapine and olanzapine also decreased β-cell insulin secretion under hyperglycaemic clamp, indicating that direct β-cell damage accompanied clozapine- and olanzapine- induced insulin resistance. Olanzapine and clozapine have been shown to decrease insulin secretion in islet β-cells (Johnson et al., 2005; Weston-Green et al., 2013). In isolated rat islets, olanzapine and clozapine inhibited cholinergic-glucose induced insulin secretion but risperidone and ziprasidone did not (Johnson et al., 2005). These results may account for the fact that diabetes is common in patients treated with clozapine and olanzapine as β-cell damage can accelerate the development of diabetes from insulin resistance.

**Figure 3:**
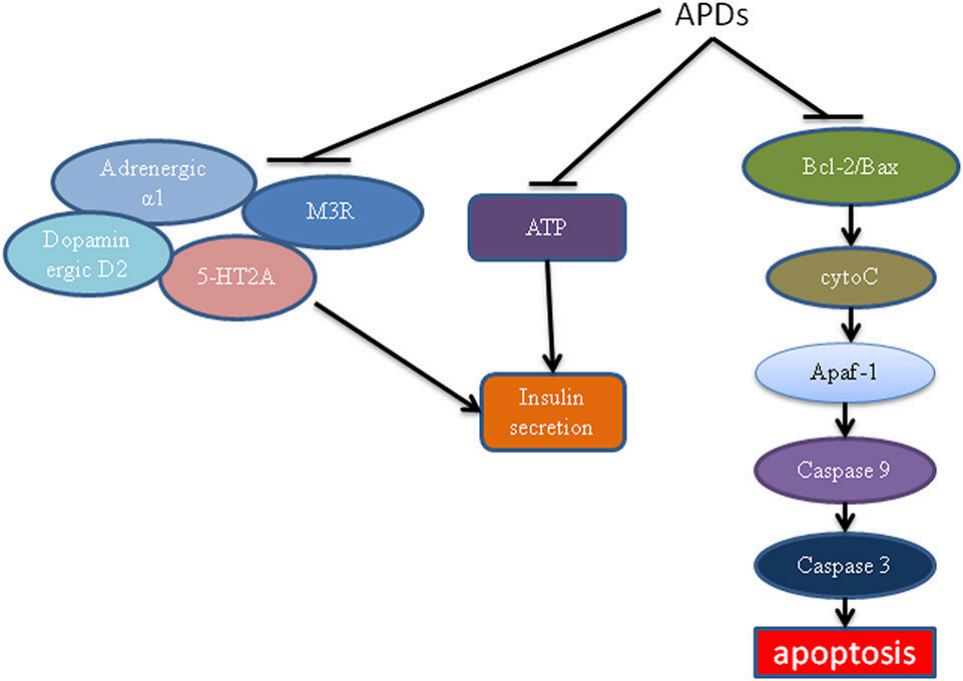
The effects of antipsychotics on β-cells. Antipsychotics can block ATP and M3, adrenergic α1 and 5-HT2A receptor-mediated insulin secretion. APDs act on the mitochondrial apoptotic pathway, leading to decreased Bcl-2 ratio, increased cytochrome c release, Apaf/caspase activation and apoptosis. APDs, antipsychotic drugs; ATP, adenosine triphosphate; M3R, muscarinic M3 receptor; Apaf, apoptotic protease activating factor.

Studies have now shown various mechanisms for APD-caused β-cell dysfunction (Figure 3). APDs can act on several β-cell receptors to cause decreased insulin secretion. Binding experiments showed that APDs can bind to various receptors including dopaminergic, histaminergic, serotonergic, adrenergic and muscarinic receptors (Guenette et al., 2013). It is known that blockade of the 5-HT2A and muscarinic M3 receptors weakens insulin response to glucose challenge, whereas antagonism at the dopamine D2 receptor increases insulin secretion (Rubí et al., 2005; Hahn et al., 2011; Weston-Green et al., 2013). Guenette et al. used a series of antagonists to screen their effects on insulin secretion including prazosin (a selective adrenergic α1 antagonist), idazoxan (a selective adrenergic α2 antagonist), SB242084 (a selective 5-HT2C antagonist), WAY100635 (a selective 5HT1A antagonist), and MDL100907 (a selective 5-HT2A antagonist). It was found that only prazosin and MDL100907 were associated with decreased insulin secretion (Guenette et al., 2013). Olanzapine and clozapine may bind to these receptors, blocking them to stimulate insulin secretion (Guenette et al., 2013). Interestingly, antagonist affinity with M3 has been demonstrated to be a main indicator of diabetes induced by APDs (Silvestre and Prous, 2005). APDs which have high binding affinity with M3 receptors, such as olanzapine and clozapine, have been shown to decrease insulin secretion (Johnson et al., 2005; Weston-Green et al., 2012b), providing clear evidence for the important role of M3 receptors in the direct effect of APDs on β-cells (Deng, 2013; Weston-Green et al., 2013).

APDs have also been linked to an increase in apoptosis of β-cells, leading to lower mass and thus decreased insulin secretion. Ozasa et al. demonstrated that APDs can act directly on β-cells to cause apoptosis (Ozasa et al., 2013). Therefore, β-cell mass is decreased, leading to decreased insulin secretion. Clozapine has also been shown to damage β-cells to cause diabetes (Best et al., 2005). The effect of APD-induced β-cell damage may be mediated by the mitochondrial apoptotic pathway (Figure 3). This pathway was regulated by proapoptotic proteins such as Bak, Bax, Bid, Bim, Bcl-associated death promoter (BAD), Noxa, and PUMA (Chen et al., 2013). It was also regulated by antiapoptotic proteins such as Bcl-2, Bcl-xL, Bcl-w, Mcl-1, and A1 (Chen et al., 2013). A changed ratio of proapoptotic proteins to antiapoptotic proteins can lead to the release of cytochrome c, resulting in promotion of the formation of active apaf (apoptotic protease activating factor)/caspase-9 complex (apoptosome), leading to caspase-3 activation and apoptosis. Contreras-Shannon et al. showed that clozapine caused mitochondrial damage in several types of insulin responding cells, including cultured mouse myoblasts (C2C12), adipocytes (3T3-L1), hepatocytes (FL-83B), and monocytes (RAW 264.7) (Contreras-Shannon et al., 2013). A proteomic study has also shown that olanzapine and clozapine cause dramatic mitochondrial protein changes in the hippocampus of rats (Ji et al., 2009). Although neither study was carried out in β-cells, they showed APDs had a direct effect on the mitochondrial apoptotic pathway. The effect of APDs on β-cell mitochondria warrants detailed investigation. It may reveal a major mechanism for APD-induced diabetes.

Decreased insulin secretion caused by APDs could also be mediated by ATP. ATP is mainly produced in mitochondria and known to regulate insulin secretion (Seino, 2012). In physiological condition, glucose metabolism increases ATP production. Accumulation of ATP in β-cells leads to closing of the K_ATP_ channels and opening of the voltage-dependent Ca^2+^ channels, resulting in Ca^2+^ influx to trigger insulin secretion (Seino, 2012). It has been shown that treatment of insulin responsive cells with clozapine reduced ATP production. It is possible that APDs also affect ATP production in β-cells.

In summary, APDs can affect the β-cell insulin secretion function. This may be mediated by several receptors particularly the M3 receptors. APDs could also cause β-cell apoptosis and decrease production of ATP. Further detailed studies will be helpful to elucidate these important mechanisms.

## Preventive and therapeutic implications

As discussed above, multiple mechanisms are involved in APD-induced diabetes including increased appetite, insulin resistance and β-cell damage. Prevention of these changes caused by APDs could be critical for medication compliance and improving clinical outcomes. To date, dozens of drugs have been trialed with some success in partly ameliorating antipsychotic-induced metabolic side-effects. Amantadine, metformin, reboxetine, sibutramine and topiramate have been shown to be effective in reducing APD-induced weight gain in Baptista et al's examination of 25 pharmacologic weight loss intervention trials (*n* = 1,221) (Baptista et al., 2008). Metformin was demonstrated to have the most promising effects on weight loss, followed by d-fenfluramine, sibutramine, topiramate, and reboxetine, in a meta-analysis of 32 placebo-controlled pharmacologic intervention trials involving 1,482 subjects (Maayan et al., 2010). Therefore, these drugs could ameliorate APD-induced insulin resistance via reducing obesity. Furthermore, the meta-analysis also showed that metformin and rosiglitazone (two antidiabetic drugs) can decrease insulin levels and insulin resistance (assessed using HOMA-IR) in patients treated with atypical APDs, even though they have no effects on fasting glucose levels (Maayan et al., 2010). However, in two studies, metformin was found to decrease fasting glucose along with decreased insulin and HOMA-IR in chronic schizophrenia patients treated with olanzapine (Baptista et al., 2006; Chen et al., 2008). In animal experiments, metformin and glyburide have been shown to reduce olanzapine-caused hyperglycaemia (Boyda et al., 2014). These results suggest that these antidiabetic drugs may directly ameliorate insulin resistance. Although metformin outperformed other agents, the current evidence is still too limited to support it as a regular adjunctive medication to control antipsychotic-induced weight gain and metabolic abnormalities (Maayan et al., 2010). Recently, a 16 weeks randomized clinical trial examined the effects of liraglutide (a glucagon-like peptide-1 [GLP-1] receptor agonist) used in conjunction with clozapine or olanzapine treatment on prediabetes and overweight/obesity in patients with schizophrenia spectrum disorders (Larsen et al., 2017). In the patients with liraglutide co-treatment, glucose tolerance was significantly improved, and particularly 63.8% prediabetic patients developed normal glucose tolerance. In addition, liraglutide induced weight loss and reduction in a number of cardiometabolic parameters including waist circumference, systolic blood pressure, visceral fat and low-density lipoprotein (Larsen et al., 2017).

Based on the key roles of histamine H1 and 5-HT2C antagonisms in APD-induced weight gain, a number of animal and/or clinical trials targeted at these receptors have be examined recently. It has been shown that co-treatment with betahistine (an H1 agonist and H3 antagonist) could partially reduce olanzapine-induced weight gain in both schizophrenia patients and animal models (Deng et al., 2012; Poyurovsky et al., 2013; Lian et al., 2014a,b, 2016). Betahistine co-treatment can also ameliorate the increased triglyceride accumulation and non-esterified fatty acids caused by olanzapine via modulating AMPK-SREBP-1 and PPARα-dependent pathways (Liu et al., 2015). A recent animal trial found that lorcaserin (a 5-HT2C-specific agonist) could not only partially reduced olanzapine-induced food intake and weight gain, but also improved glucose tolerance in female mice (Lord et al., 2017). In future, the major effort could be the elucidation of the mechanisms for APD-induced diabetes, particularly APD-induced β-cell damage. It is only the beginning to realize the important role of β-cells in APD-induced diabetes. Since APDs may decrease insulin secretion through blocking M3 receptors on β-cells (Johnson et al., 2005; Weston-Green et al., 2013), it is worth to trial whether a specific M3 agonist could mitigate APD-induced diabetes. Drugs or phytochemicals that can protect β-cells may be used for the prevention of APD-induced diabetes. For example, resveratrol and curcumin have been demonstrated to be effective to protect β-cells in streptozotocin-induced damage (Su et al., 2006; Meghana et al., 2007; Palsamy and Subramanian, 2010), which may be used in APD-induced β-cell damage.

## Conclusions

APDs are associated with diabetes and hyperglycaemic emergency. This could be mediated by multiple mechanisms. APDs can inhibit the insulin signaling pathway in insulin sensitive cells such as muscle cells, hepatocytes and adipocytes to cause insulin resistance. APD-induced obesity can result in high levels of FFA and inflammation, which can cause insulin resistance as well. More importantly, APDs can cause direct damage to β-cells. This accounts for the rapid development of diabetes and severe diabetic complications. As multiple mechanisms are involved in APD-induced diabetes, preventive and therapeutic approaches may need to combine agents counteracting different mechanisms. Among them prevention of β-cell damage is of particular importance as diabetes would not happen without β-cell damage.

## Author contributions

JC and CD conceived and outlined the manuscript. JC wrote the first draft of the manuscript. CD, X-FH, RS, and CC contributed to manuscript writing, editing, synthesis of previous literature.

### Conflict of interest statement

The authors declare that the research was conducted in the absence of any commercial or financial relationships that could be construed as a potential conflict of interest. The reviewer IG-G and handling Editor declared their shared affiliation.
